# Obituary

**Published:** 1890-06

**Authors:** 


### Obituary.

Died, at Compton Creek, Fayette County, Ohio, April 18th, 1890, of heart disease, Dr. Luman Birch Welsch, D.D.S., of

Wilmington, Ohio. Dr. Welsch had been forty-one years in the practice of dentistry in Wilmington, and as a professional man he occupied an enviable position. In his early life he attained an academic education ; afterward he studied medicine, and practiced in Knox County, Ohio. After being thus engaged for some time he decided to take some specialty of medical practice, and finally chose that of dentistry, which required great effort to acquire knowledge necessary under the disadvantages of forty years ago. At that time every dentist had to manufacture, more or less, the instruments he used.

The degree of Doctor of Dental Surgery was conferred on him in 1872, by the Ohio College of Dental Surgery. He was very skilful in the art and practice of his profession, for which he had a great love; but while this is true, he devoted much attention to collateral subjects. As a geologist he had a wide reputation; to this he devoted much of his leisure time. He collected a large cabinet of geological specimens. He took much interest in the works of the Ancient Mound Builders; examined many of the mounds and accumulated numerous relics from them.

He was a student of nature, and his spare time was almost wholly spent out-of-doors. He was fond of his gun and rod, and for many years has been regarded as an authority on all questions of the field or stream.

On the day of his death he and a friend left home for a day's fishing, and in the afternoon of that day suddenly he was prostrated and his spirit took its flight from the banks of the beautiful stream where he was engaged in his chosen sport.

He was joined in marriage to Cloe E. Griffin, of Fittsville, Huron County, Ohio, October 29th, 1848. January following they moved to Wilmington, where they have since had their home, and for over forty years his residence and office have been in the same building. To Dr. Welsch and wife were born three children only one of whom, namely, Dr. Charles Welsch, D.D.S., survives him.

His love of nature and out-door life were great, so we can but think it fitting that the happy spirit should lay down its tenement of clay on the green bank of the beautiful stream of Compton Creek, under the drooping willow, on the beautiful day, April 18, 1890.

